# Peanut lipids influence the response of bronchial epithelial cells to the peanut allergens Ara h 1 and Ara h 2 by decreasing barrier permeability

**DOI:** 10.3389/fmolb.2023.1126008

**Published:** 2023-02-08

**Authors:** Chiara Palladino, Isabella Ellinger, Tanja Kalic, Piotr Humeniuk, Davide Ret, Vanessa Mayr, Christine Hafner, Wolfgang Hemmer, Karin Hoffmann-Sommergruber, Eva Untersmayr, Merima Bublin, Christian Radauer, Heimo Breiteneder

**Affiliations:** ^1^ Institute of Pathophysiology and Allergy Research, Center for Pathophysiology, Infectiology and Immunology, Medical University of Vienna, Vienna, Austria; ^2^ Department of Dermatology, University Hospital St. Pölten, Karl Landsteiner University of Health Sciences, St. Pölten, Austria; ^3^ Division of Macromolecular Chemistry, Institute of Applied Synthetic Chemistry, Vienna University of Technology, Vienna, Austria; ^4^ Karl Landsteiner Institute for Dermatological Research, St. Pölten, Austria; ^5^ FAZ-Floridsdorf Allergy Center, Vienna, Austria

**Keywords:** peanut allergy, epithelial cells, lipids, allergic sensitization, peanut allergens

## Abstract

**Background:** Peanut-allergic individuals react upon their first known ingestion of peanuts, suggesting sensitization occurs through non-oral exposure. Increasing evidence suggests that the respiratory tract is a probable site for sensitization to environmental peanuts. However, the response of the bronchial epithelium to peanut allergens has never been explored. Furthermore, food matrix-derived lipids play an important role in allergic sensitization.

**Objective:** To contribute to a better understanding of the mechanisms of allergic sensitization to peanuts *via* inhalation, by exploring the direct effect of the major peanut allergens Ara h 1 and Ara h 2 and peanut lipids on bronchial epithelial cells.

**Methods:** Polarized monolayers of the bronchial epithelial cell line 16HBE14o- were stimulated apically with peanut allergens and/or peanut lipids (PNL). Barrier integrity, transport of allergens across the monolayers, and release of mediators were monitored.

**Results:** Ara h 1 and Ara h 2 impacted the barrier integrity of the 16HBE14o- bronchial epithelial cells and crossed the epithelial barrier. Ara h 1 also induced the release of pro-inflammatory mediators. PNL improved the barrier function of the cell monolayers, decreased paracellular permeability and reduced the amount of allergens crossing the epithelial layer.

**Conclusion:** Our study provides evidence of the transport of Ara h 1 and Ara h 2 across the airway epithelium, of the induction of a pro-inflammatory milieu, and identifies an important role for PNL in controlling the amount of allergens that can cross the epithelial barrier. These, all together, contribute to a better understanding of the effects of peanuts exposure on the respiratory tract.

## 1 Introduction

Peanut allergy is a serious disease affecting 1%–3% of the population in Western countries and is responsible for the majority of severe reactions and death cases related to food allergy (Pouessel et al., 2018; Yue et al., 2018; Baseggio Conrado et al., 2021). Compared to other food allergies, peanut allergy more often persists for a lifetime and represents an economic and health burden to allergic patients (and their caregivers) (Cannon, 2018; Sicherer and Sampson, 2018). Up to now, 17 peanut allergens have been officially recognized by the WHO/IUIS Allergen Nomenclature Sub-Committee (http://www.allergen.org/), and among those, the seed storage proteins Ara h 1 and Ara h 2 are major allergens and primary sensitizers associated with clinical severity (Vereda et al., 2011; Palladino and Breiteneder, 2018). The vast majority of peanut-allergic subjects react upon their first known ingestion of peanuts (Sicherer et al., 1998), suggesting that sensitization occurs through non-oral exposure routes. Over the past decade, cutaneous sensitization to food allergens has become well accepted showing that early cutaneous exposure to food protein through a disrupted skin barrier leads to allergic sensitization (Du Toit et al., 2016). However, there is increasing evidence that other environmental peanut exposure routes, such as inhalation, also play an important role (Smeekens et al., 2019; Kulis et al., 2021).

Several case reports have suggested potential sensitization to food antigens such as millet, buckwheat, lupine seed flour, egg, and sunflower seed through the airway and subsequent anaphylaxis after oral ingestion of the food (Jungewelter et al., 2020; Kotachi et al., 2020; Kulis et al., 2021). Indoor dust acts as an adjuvant to promote sensitization to pollens, pet dander, dust mite, and peanuts (Smeekens et al., 2019). It has been described that airborne peanut particles caused allergic reactions in peanut-allergic individuals within the environment of an airplane (Sicherer et al., 1999). Also, it has been demonstrated that peanut protein presents in household dust is biologically active (Brough et al., 2013) and that environmental peanut exposure increases the risk of peanut sensitization in children (Brough et al., 2018). Despite the growing body of evidence suggesting that exposure to food allergens in the respiratory tract is a possible route of sensitization (Kulis et al., 2021), data exploring the direct effect of peanut allergens on respiratory epithelium are still lacking.

Even though the underlying mechanisms of allergic sensitization have not been fully elucidated, it can be assumed that the first step of the sensitization process is the entry of the allergen across epithelial surfaces (airways, gastrointestinal tract, and skin). For this reason, epithelial cells play a crucial role in this process, as they not only represent a physical barrier but interact and respond to allergens stimuli by mounting an innate immune response (Lambrecht and Hammad, 2014). Allergens can interact with epithelial cells by activating receptors on the cells’ surface (Hammad et al., 2009) or by direct cleavage of tight junctions (TJ) proteins favoring allergen penetration through epithelial layers (Wan et al., 1999) and gaining access to dendritic cells. How peanut allergens interact with airway epithelial cells and how this might affect the sensitization process is currently unknown. Moreover, increasing evidence points toward a role for lipids from the allergen source in the allergic sensitization process (Bublin et al., 2014; Del Moral and Martínez-Naves, 2017; Hopkins et al., 2022). Our group previously showed that peanut lipids induced a pro-inflammatory response in human keratinocytes (Palladino et al., 2018), which could provide Ara h 1 and Ara h 2 with the right milieu for the orchestration of Th2 immune responses leading to sensitization. Furthermore, it has been demonstrated that food processing, and specifically roasting, impacts peanut allergenicity (Maleki et al., 2000; Verhoeckx et al., 2015).

In this study, we aimed to contribute to the understanding of the mechanisms of allergic sensitization to peanuts *via* inhalation. Hence, in this study we explored the effect of the major peanut allergens Ara h 1 (7S globulin) and Ara h 2 (2S albumin) on the airway epithelium by direct apical stimulation of the polarized 16HBE14o- cells, a well-established model of the bronchial epithelium (Georas and Rezaee, 2014). We evaluated the barrier integrity, the paracellular transport of allergens, and the associated release of cytokines. We also hypothesized a role for PNL in allergic sensitization to peanuts and studied their impact on the bronchial epithelial cell monolayers. Finally, we investigated whether various lipid preparations make a difference in the effect on the respiratory epithelium. To the best of our knowledge, this study is the first exploring the effect of peanut allergens and lipids on the bronchial epithelial cells, and the first to demonstrate that Ara h 1 and Ara h 2 are internalized by bronchial epithelial cells and transported through the epithelial monolayers in an immunologically active form. Moreover, PNL modified the physical barrier properties and reduced the amount of allergens transported across the epithelial monolayers.

## 2 Materials and methods

### 2.1 Bronchial epithelial cell culture

The 16HBE14o- cell line, derived from human bronchial epithelial cells, was cultured in minimum essential medium (MEM) supplemented with 10% fetal bovine serum (FBS) (Gibco, Thermo Fisher Scientific, MA, United States), 100 U/mL penicillin and 100 μg/mL streptomycin (Sigma–Aldrich, MO, United States). Cells were grown in tissue culture flasks pre-coated with human fibronectin and bovine collagen (BD Biosciences, CA, United States) and passaged when reaching 80%–90% confluence. Experiments were performed with cells within four passage numbers using antibiotic-free MEM containing 1% FBS. If not specified differently, 4 × 10^5^ cells/well were seeded on 12-mm permeable filter supports (0.4 µm pore size; Transwell^®^, Corning, Thermo Fisher Scientific, MA, United States). 0.5 and 1.5 mL of medium were added to the upper and lower chamber, respectively. The formation of cell monolayers was monitored continuously by measurement of the transepithelial electrical resistence (TEER).

### 2.2 Isolation and purification of Ara h 1 and Ara h 2

Natural peanut allergens Ara h 1 and Ara h 2 were purified and characterized as previously described (Palladino et al., 2018). Endotoxin contamination of both peanut allergens was removed by EndoTrap columns (Hyglos GmbH, Bernried am Starnberger See, Germany). Remaining endotoxin measured by the LAL test (EndoZyme, Hyglos GmbH, Bernried am Starnberger See, Germany) was below 10 EU/mg. The concentrations of the allergens were determined using the Pierce BCA Protein Assay Kit (Thermo Fisher Scientific, MA, United States).

### 2.3 Lipids

PNL were isolated from commercially available roasted peanuts as previously described (Palladino et al., 2018). The same protocol was used to extract lipids from raw peanuts (PNL-Raw), and Timothy grass (*Phleum pratense*) pollen (GPL). Lipids were also extracted from peanuts boiled in water for 30 min (PNL-B). PNL and GPL were fractionated as follows: the lipid extracts diluted in chloroform were loaded onto thin-layer chromatography (TLC) plates (TLC AI foil, Sigma–Aldrich, MO, United States). TLC was conducted as previously described (Palladino et al., 2018), and visualized by iodine (Sigma–Aldrich, MO, United States). Once separated, fractions were scratched from the plate, and lipids were re-extracted with 10 mL of MeOH:CHCl_3_ 1:2. Every fraction was evaporated by a nitrogen stream and weighed. Lipids resulted negative when tested for the presence of innate immune system contaminants by means of a cellular assay with the reporter cell lines THP1-XBlue and THP1-XBlue-MD2-CD14 (InvivoGen, San Diego, CA, United States). Glyceryl trioleate and oleic acid were purchased from Sigma–Aldrich, MO, United States and used as control for lipids. PNL and GPL total extracts, as well as individual fractions and synthetic lipids were applied to 16HBE14o- cells at final concentrations of 100 μg/mL, or at a varying concentrations if specified, after 5 min of sonication in the medium.

### 2.4 Monitoring permeability of cell monolayers treated with allergens and lipids

TEER was measured using an epithelial voltohmmeter (EVOM2, World Precision Instruments, Thermo Fisher Scientific, MA, United States). The resistance of empty filters (130 Ω cm^2^ on average) was subtracted from TEER measurements done on filter-grown cells. TEER was calculated using the equation: (TEER sample − TEER blank) × surface area (cm^2^). After seeding cells, TEER was monitored twice a day, until the resistance exceeded 700 Ω cm^2^. This value was usually reached approximately 50 h after seeding. The TJ protein *zonula occludens* (ZO)-1 was visualized by immunofluorescence microscopy to confirm TJ formation (data not shown). Experiments were performed within 16 h after reaching 700 Ω cm^2^. All reagents were added to the upper chamber of the permeable filter insert, thus were applied to the apical membrane of the cells. Allergens and lipids were used at a concentration of 100 μg/mL. Lower doses of allergens or lipids (25 and 50 μg/mL) were used to evaluate dose-response effects. Untreated cells were used as controls. Positive controls for barrier disruption were the proteolytic enzyme papain from *Carica papaya* at 75 μg/mL (Kouzaki et al., 2009) and a combination of TNF-α (50 ng/mL) and IL-1β (20 ng/mL) (Kimura et al., 2009; Hardyman et al., 2013).

### 2.5 Analysis of paracellular permeability of 16HBE14o- monolayers

Changes in the paracellular permeability of cell monolayers were investigated by measuring the passage of fluorescein isothiocyanate (FITC)-labeled dextran (FD) with different molecular weights across these layers. For this purpose, cells were cultured in phenol-red free minimum essential medium (Lonza, Switzerland) on Transwell^®^ filters as described above. FDs of three different sizes (molecular mass 4, 10, and 70 kDa, Sigma–Aldrich, MO, United States) were applied to the upper compartment at a concentration of 2 mg/mL alone or together with PNL and/or allergens (100 μg/mL), and aliquots of the basolateral medium were collected 2, 6, and 16 h after the treatment. The fluorescence intensity (excitation: 490 nm; emission: 520 nm) of collected samples was measured by an Infinite 200 Pro plate reader (Tecan, Switzerland) and fluorescence values measured in media obtained from non-treated cells were subtracted. The amount of FD in the basolateral medium was calculated using a standard curve. As known disruptors of TJ, 0.5 mM H_2_O_2_ (Sigma–Aldrich, MO, United States), and 5 mM ethylene glycol-bis(β-aminoethyl ether)-N,N,N′,N′-tetraacetic acid (EGTA) were applied (Olson et al., 2009). Before EGTA treatment, cells were pre-washed with phosphate-buffered saline (PBS) to remove all traces of FBS.

### 2.6 Confocal microscopy of 16HBE14o- monolayers

16HBE14o- cells (3.4 × 10^4^ cells/well) were seeded in complete MEM onto human fibronectin and bovine collagen (BD Biosciences) coated round 12 mm coverslips (Thermo Fisher Scientific), placed into a 24-well plate and incubated for 50 h at 37°C and 5% CO_2_. Cells were washed with PBS and incubated for 1 h in fresh serum-free and antibiotic-free MEM. Ara h 1 and Ara h 2 were labeled with AlexaFluor488^®^ (Thermo Fisher Scientific) following the manufacturer’s instructions. 100 μg/mL of fluorescent-labeled allergens were applied to the cells for 1 h at 37°C. In all experiments, untreated cells were used as a negative controls. After treatment, the monolayers were washed with cold PBS, fixed with 4% formaldehyde in PBS, and then permeabilized using blocking buffer (1% bovine serum albumin in PBS) supplemented with 0.5% saponin (Carl Roth, Germany). To determine the degree of confluence and monolayer formation, 16HBE14o- cells were incubated for 1 h at RT with an anti-ZO-1 antibody (PA5-28869, Thermo Fisher Scientific) diluted 1:500 in blocking buffer, followed by incubation for 1 h at RT with an anti-rabbit IgG AlexaFluor568^®^-conjugated antibody (A11011, Invitrogen, Thermo Fisher Scientific) diluted 1:2,000 in blocking buffer. To visualize late endosome/lysosome localization, the lysosomal-associated membrane protein-2 (LAMP-2) was detected with a primary antibody (BD555803, BD Biosciences) diluted 1:50 in blocking buffer, followed by incubation with an anti-rabbit AlexaFluor568^®^-conjugated antibody (A11011, Invitrogen, Thermo Fisher Scientific) diluted 1:2,000 in blocking buffer for 1 h at RT. Nuclei were stained with 5 mM DRAQ5™, according to the manufacturer’s instructions (Abcam, United Kingdom). Inverted coverslips were mounted on microscope glass slides using Fluoromount-G^®^ (Southern Biotech, AL, United States) and allowed to dry overnight in the dark. Confocal images of samples were acquired using an UltraVIEW ERS Rapid Confocal Imager (Perkin-Elmer, Waltham, MA, United States) connected to a Zeiss Axiovert 200 microscope fitted with a 63×/1.4 oil objective lens (Plan-Apochromat, Zeiss, Jena, Germany). Alexa Fluor-488 and -568 fluorophores, as well as DRAQ5, were excited at 488, 568, and 647 nm, respectively, using a 488/548/647 multiline argon/krypton laser. Pictures were digitized and processed by Volocity software (Version 5.5, Perkin Elmer).

### 2.7 Quantification of trans-epithelial allergen transport by immunoassays

The peanut allergens Ara h 1 and Ara h 2, alone or in combination with PNL were applied apically to 16HBE14o- cells, and aliquots of the basolateral medium were collected 2, 6, and 16 h thereafter. The amounts of allergens in the basolateral medium were quantified by enzyme-linked immunosorbent assay (ELISA) from a standard curve. Anti-Ara h 1 (MA-2F7) and anti-Ara h 2 (MA1-4C) antibodies were obtained from InBio, Cardiff, United Kingdom and used as capture antibodies coated to a 96-well plate (Maxisorp Immunoplate, Nunc, Thermo Fisher Scientific). Peanut allergens detection was performed with a pool of sera from three peanut-allergic individuals recruited at the FAZ-Floridsdorf Allergy Center (Vienna, Austria) and the Department of Dermatology of the University Hospital St. Pölten (St. Pölten, Austria). IgE bound to the allergens was detected by an alkaline phosphatase-conjugated anti-human IgE antibody (BD Bioscience, CA, United States).

Additionally, we also performed the detection of Ara h 1 and Ara h 2 in the basolateral medium by Western blot (WB). Samples collected 16 h after treatment with Ara h 2 alone (100 μg/mL) or in combination with PNL (100 μg/mL) were concentrated by ultracentrifugation using an Amicon^®^ Ultra Centrifugal Filter with a 3 kDa molecular weight cut-off (Merck Millipore, Burlington, MA, United States). Samples were separated by 12% SDS-PAGE under reducing condition and then electrotransferred onto a polyvinylidene difluoride (PVDF) membrane. After blocking with Tris-buffered saline (TBS) containing 0.5% Tween20 and 1% bovine serum albumin (Carl Roth, Germany), the membrane was incubated with a rabbit polyclonal anti-Ara h 2 antibody produced by immunization of rabbits with natural Ara h 2 at the animal care unit of the Department of Toxicology (Slovak Medical University, Bratislava, Slovakia). Rabbits were kept according to the local guidelines for animal welfare. Bound antibodies were detected by an anti-rabbit horseradish peroxidase-linked IgG antibody (Cell Signaling Technology, Danvers, MA, United States). Reactive bands were visualized by incubating the membrane with Luminol chemiluminescent substrate (Santa Cruz Biotechnology, Dallas, TX, United States) and exposing the membrane to a film.

### 2.8 Quantification of cytokines released from the basolateral side of the cell monolayer

Samples of basolateral medium were collected 16 h after stimulation of cells with allergens alone or in combination with PNL. Concentrations of IL-6, IL-8, and CCL-2 were determined using the HCYTOMAG-60K kit (Merck Millipore, MA, United States) and a Luminex 200 System (Luminex’s-Hertogenbosch, Netherlands).

### 2.9 NMR spectroscopy of lipids

^1^H-Nuclear magnetic resonance (NMR) spectroscopy of lipids (PNL, PNL-Raw, and PNL-B) was performed with a Bruker Avance DRX-400 MHz FT-spectrometer. Each sample (1–3 µL) was dissolved in CDCl_3_ (0.7 mL) and then transferred into an NMR glass tube. The proton spectra were recorded with 16 scans. All spectra were processed using MestReNova Software (Mestrelab Research, Spain). Chemical shifts are displayed in ppm. All spectra are referenced to the chloroform peak at 7.26 ppm (Fulmer et al., 2010).

### 2.10 Statistical analysis

Statistical analysis was performed using GraphPad Prism software version 6 (GraphPad Software, CA, United States). When not stated otherwise, the statistical significance was calculated using two-way ANOVA, followed by the Tukey test to correct for multiple comparisons. *p*-values below 0.05 were regarded significant: *p < 0.05; **p < 0.01; ***p < 0.001; ****p < 0.0001.

## 3 Results

### 3.1 The barrier function of 16HBE14o- cell monolayers is impaired when exposed to peanut allergens but improved by peanut lipids

**FIGURE 1:**
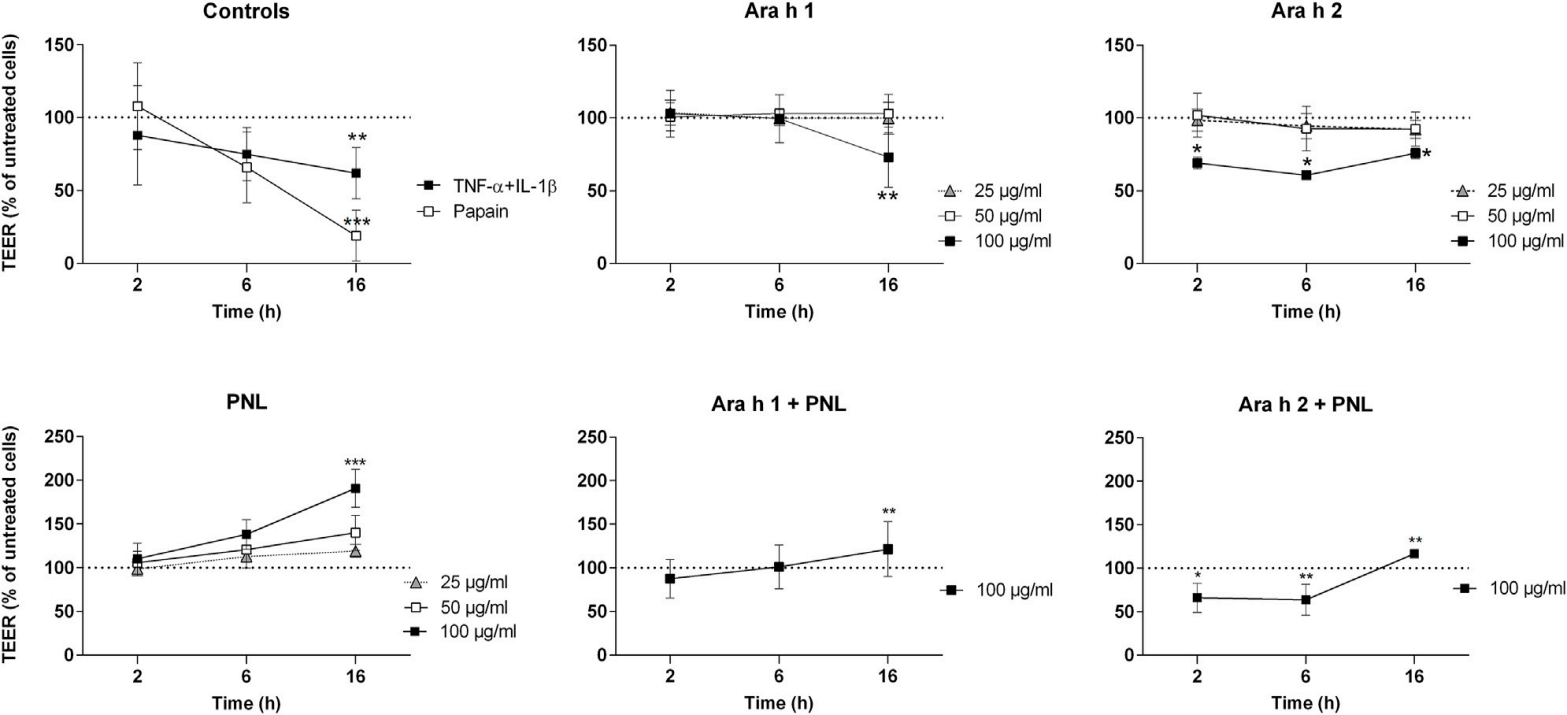
Impact of peanut allergens and lipids on the permeability of polarized 16HBE14o- cell monolayers. Treatments were applied apically to cell monolayers and the TEER was measured 2, 6, and 16 h after treatment. Positive controls were papain and the combination of TNF-α and IL-1β. Ara h 1, Ara h 2, and PNL were used at different concentrations (25, 50, and 100 μg/mL). Cells were also treated with a combination of Ara h 1 + PNL and Ara h 2 + PNL (the concentration of the single component was 100 μg/mL). TEER values of untreated cells were set as 100% and are indicated by dashed lines. Data show the mean ± SD of three independent experiments. *p < 0.05, **p < 0.01, ***p < 0.001.

We characterized the effect of the peanut allergens Ara h 1 and Ara h 2, and of PNL on the barrier function of polarized 16HBE14o- cells (Figure 1). The barrier properties were monitored by TEER measurement. TNF-α + IL-1β and papain, positive controls for the barrier damage, caused a drastic decrease of TEER compared to untreated cells (TNF-α + IL-1β *p* < 0.01; papain *p* < 0.001). Only the highest concentration of Ara h 1 (100 μg/mL) affected the permeability of the cells after 16 h of treatment (*p* < 0.01 vs. untreated). Ara h 2 at the highest concentration of 100 μg/mL caused a significant TEER decrease already after 2 h of exposure (*p* < 0.05 vs. untreated), which then remained constant for the duration of the treatment. An opposite effect was seen upon the application of PNL to cell monolayers. A dose-dependent increase of the TEER started already at 6 h of treatment. At the highest concentration of PNL (100 μg/mL), TEER values almost doubled compared to untreated cells at 16 h (*p* < 0.001).

We next examined the effect of a combination of allergens and PNL on the barrier function of cell monolayers. After 16 h, a concentration of 100 μg/mL Ara h 1 in the presence of 100 μg/mL PNL did not reduce TEER as seen for Ara h 1 alone, but instead, the TEER increased significantly (Ara h 1 + PNL vs. Ara h 1 *p* < 0.01). While treatment with 100 μg/mL Ara h 2 in the presence of PNL caused a drop of the TEER after 2 and 6 h as seen for Ara h 2 alone, the presence of PNL induced an increase of the TEER at 16 h. TEER values after Ara h 2 + PNL treatment were significantly lower than after PNL treatment at 16 h (*p* < 0.01) and significantly higher than after Ara h 2 treatment (*p* < 0.01).

### 3.2 Treatment of 16HBE14o- cell monolayers with PNL decreases the paracellular diffusion of dextran

We examined whether peanut allergens and PNL affect the paracellular permeability in 16HBE14o- cell monolayers by measuring the permeability for FITC-dextrans of different molecular masses. The positive controls H_2_O_2_ and EGTA caused a dramatic decrease in the TEER already after 2 h of treatment (Supplementary Figure S1). Treatments of cells with FD4, FD10, or FD70 in combination with PNL, Ara h 1 alone or with PNL, and Ara h 2 alone or with PNL resulted in TEER values similar to those presented in Figure 1, indicating that FDs *per se* had no specific impact on the TEER.

**FIGURE 2:**
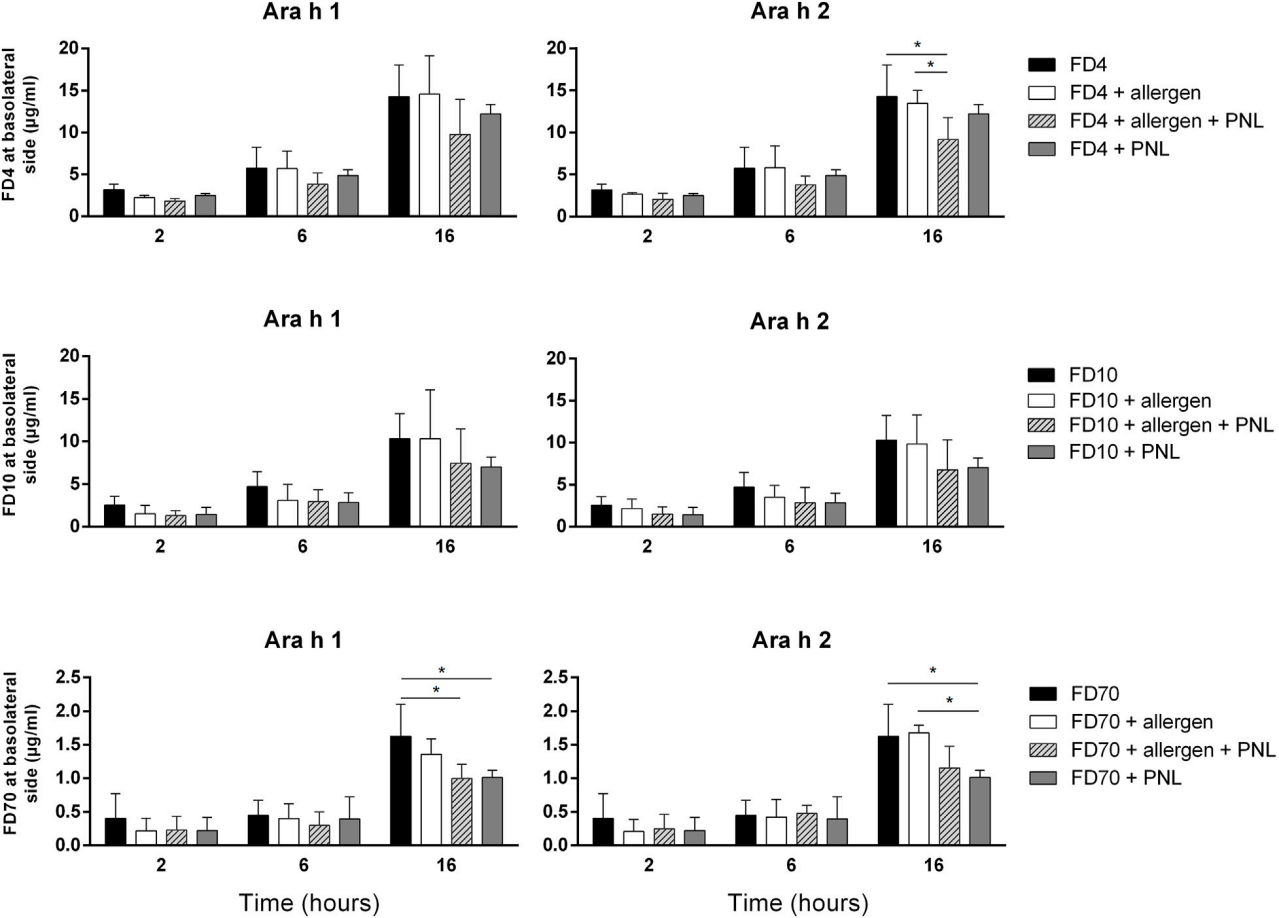
Paracellular permeability assay. Transport of FD4, FD10, or FD70 through the cell monolayer, alone or in combination with Ara h 1, Ara h 2 and/or PNL was quantified in basolateral cell culture medium by fluorometry. Data (mean ± SD of three experiments) show levels of FD after 2, 6, and 16 h of apical exposure.

Afterward, we quantified FD4, FD10, or FD70 passing through the cell monolayers upon treatment with peanut allergens and/or PNL after 2, 6, and 16 h (Figure 2). Neither Ara h 1 nor Ara h 2 alone impacted the amount of individual FDs in the basolateral cell culture media compared to cells exposed only to FD alone. In contrast, less FD4, FD10, or FD70 was detected in the basolateral media of cells treated with PNL in the absence or presence of allergens, indicating that PNL reduced paracellular permeability. PNL decreased the paracellular permeability of dextran already after 2 h upon cell stimulation but became more evident after 6 and then 16 h of stimulation. Although statistical significance was only observed for PNL + FD4 and PNL + FD70 at the latest time point measured, PNL + FD10 showed a similar trend. As controls, we also measured the concentrations of FD4, FD10, or FD70 at the basolateral side upon stimulation with EGTA and H_2_O_2_, which allowed a significant amount of FD4, FD10, and FD70 to cross the barrier as compared to FD alone (Supplementary Figure S2).

### 3.3 Quantification of trans-epithelial allergen migration by immunoassays and confocal microscopy analysis of internalized peanut allergens

**FIGURE 3:**
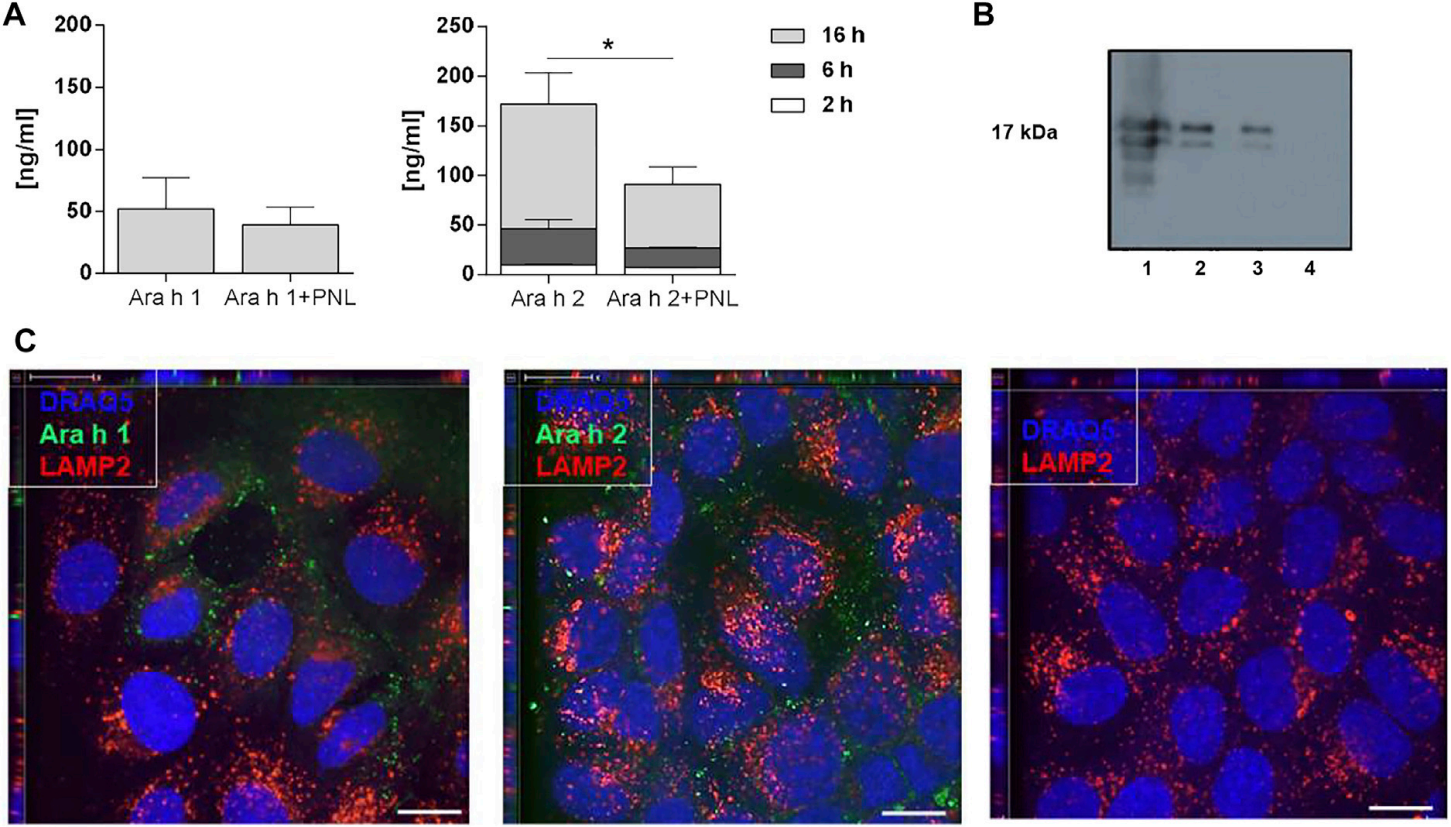
Ara h 1 and Ara h 2 are transferred to the basolateral compartment and internalized by 16HBE14o- cells. **(A)** Quantification of peanut allergens in the basolateral medium 2, 6, and 16 h after apical addition of Ara h 1 and Ara h 2 alone or combined with PNL. Allergens were quantified by ELISA using IgE from peanut-allergic individuals. Data of three independent experiments are presented as mean ± SD. **(B)** Detection of Ara h 2 in the basolateral medium by WB 16 h after apical addition of Ara h 2 ± PNL. Lane 1: purified Ara h 2 (0.5 µg); loading control; Lane 2: Ara h 2; Lane 3: Ara h 2 + PNL; Lane 4: basolateral cell culture medium of untreated cells. **(C)** Confocal immunofluorescence microscopy images of 16HBE14o- cell monolayers. Allergens (green) were internalized for 1 h at 37°C. In the negative control, no allergen was present. The late endosome/lysosome marker LAMP2 is in red and nuclei in blue. Scale bar: 10 µm.

In a next step, we investigated the ability of peanut allergens to cross the cell monolayer in the absence and presence of PNL. After the addition of allergens in presence or absence of PNL to the apical compartment, samples of basolateral medium were collected 2, 6, and 16 h after stimulation, and Ara h 1 and Ara h 2 were quantified by ELISA (Figure 3A). After 16 h of exposure, Ara h 1 was detected in the basolateral medium. The simultaneous presence of PNL caused a slight but non-significant reduction of Ara h 1 in the basolateral medium. Ara h 2 was already detected after 2 h. Cells treated with Ara h 2 in the presence of PNL exhibited significantly reduced amounts of Ara h 2 in their basolateral media compared to those treated with allergens alone (*p* < 0.05). The difference in the amounts of Ara h 2 detected at the basolateral side of the cell monolayers treated with either Ara h 2 alone or with PNL was also visualized by WB (Figure 3B, lanes 2 and 3). Positive (lane 1) and negative (lane 4) controls for WB were purified Ara h 2 and basolateral medium of untreated cells, respectively. We did not detect Ara h 1 by WB.

Molecules may cross epithelia *via* a paracellular or transcellular route. We investigated the endocytosis of allergens by confocal microscopy after adding fluorescently labeled Ara h 1 or Ara h 2 to the apical side for 1 h at 37°C (Figure 3C). The allergens (green) were internalized by the cells and were visible at the nuclei levels. Neither Ara h 1 nor Ara h 2 co-localized with the late endosome/lysosome marker LAMP2 (red), indicating that the allergens did not enter the lysosomal pathway following internalization.

### 3.4 Analysis of the cellular cytokine response

**FIGURE 4:**
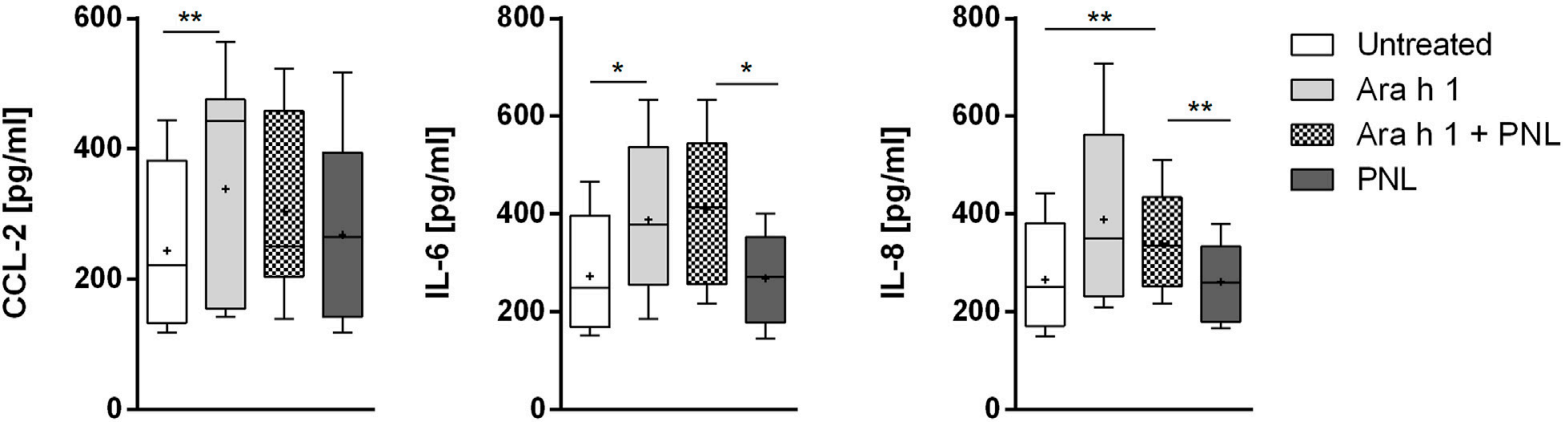
Induction of cytokines release. Cell monolayers were treated apically with Ara h 1 ± PNL, and the concentrations of CCL2, IL-6, and IL-8 released into the basolateral medium were measured after 16 h.

We checked if peanut allergens would influence the release of cytokines and mediators into the basolateral compartment of the 16HBE14o- monolayer (Figure 4). Ara h 1 increased the release of the pro-inflammatory cytokines CCL2, IL-6, and IL-8. Treatment of cells with PNL alone did not affect the release of cytokines compared to untreated cells. Similarly, when PNL were co-administered with the allergen. Ara h 2 did not increased the release of CCL-2, IL-6, and IL-8 (Supplementary Figure S4).

### 3.5 The effect of lipids on the epithelial barrier function is not unique for peanut lipids

**FIGURE 5:**
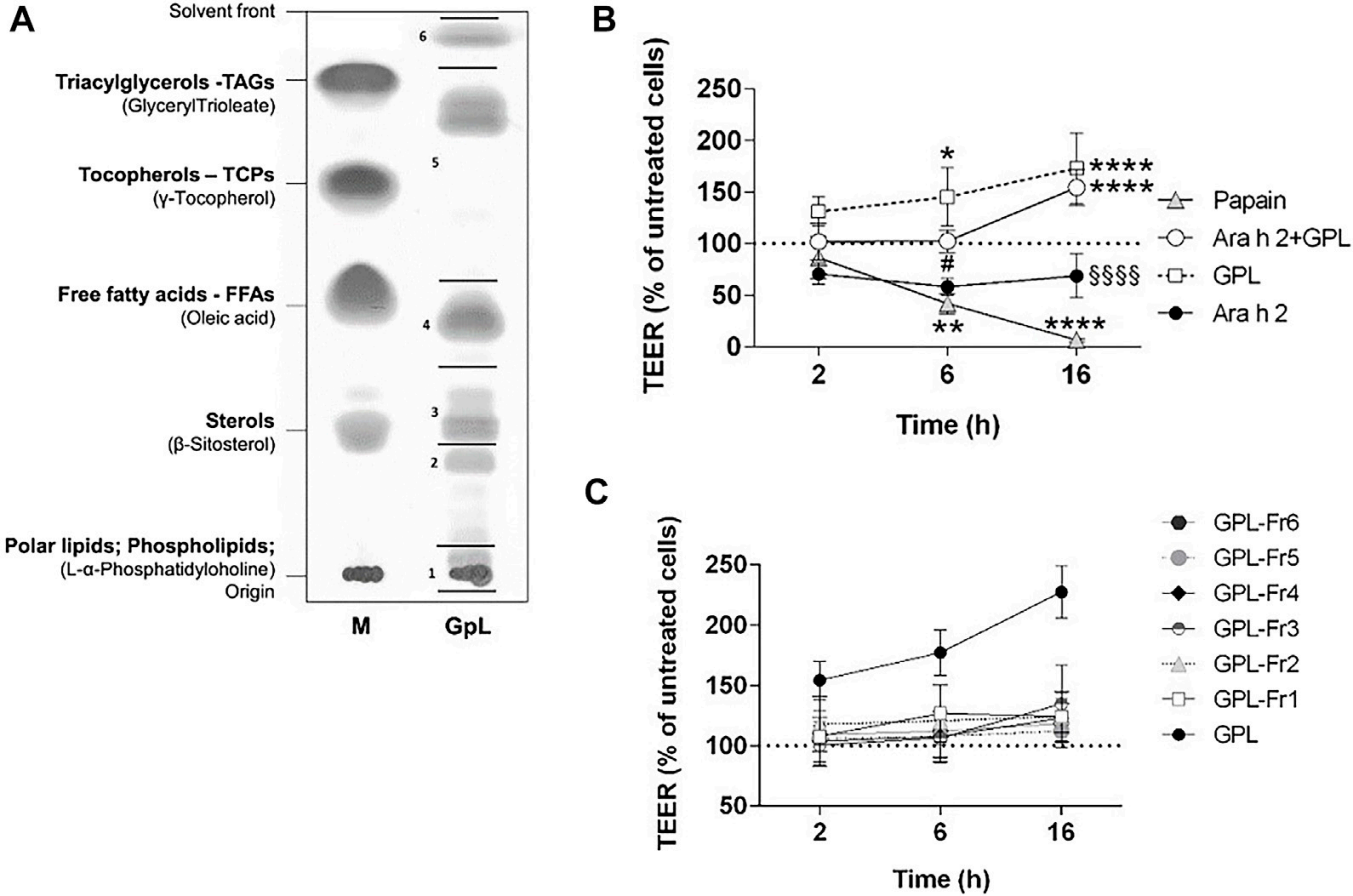
Impact of GPL and GPL-fractions on the TEER permeability of polarized 16HBE14o- cell monolayers. **(A)** TLC of GPL separated into major lipid classes in parallel to a lipid marker (M). The lipid samples loaded as markers are given in brackets, the corresponding major lipids classes are indicated in bold. GPL were separated into fractions indicated by black lines and numbers. **(B)** Cell monolayers were treated apically with GPL, Ara h 2, and their combination. TEER values were measured after 2, 6, and 16 h and results are depicted as percentage of TEER of untreated cells (dashed line), papain was used as a positive control. Data represent the mean ± SD of three experiments. *p < 0.05, **p < 0.01, ***p < 0.001. (*) indicates significance compared to untreated cells (negative control); (#) indicates significance compared to GPL; (§) indicates significance compared to Ara h 2 + GPL. **(C)** GPL fractions together with GPL, were applied to the apical compartments of cell monolayers and TEER was measured after 2, 6, and 16 h. Results are shown as the percentage of TEER of untreated cells (dashed line). Data represent the mean ± SD from three experiments.

We questioned whether the effect observed for PNL on the airway epithelium could be also triggered by lipids from a typical source of aeroallergens. Therefore, we isolated lipids from Timothy grass pollen (GPL) (Figure 5A) and apically stimulated 16HBE14o- epithelial cells with GPL alone or in combination with Ara h 2. GPL increased the TEER of 16HBE14o- cells and their effect was very robust compared to untreated cells (GPL 6 h *p* < 0.05; 16 h *p* < 0.0001) (Figure 5B). Moreover, the decrease of the TEER after 2 h of treatment with Ara h 2 was counteracted by GPL (16 h Ara h 2 + GPL vs. Ara h 2 *p* < 0.0001). Overall, GPL showed a similar, and even stronger, impact than PNL on the barrier function of 16HBE14o- cells.

**FIGURE 6:**
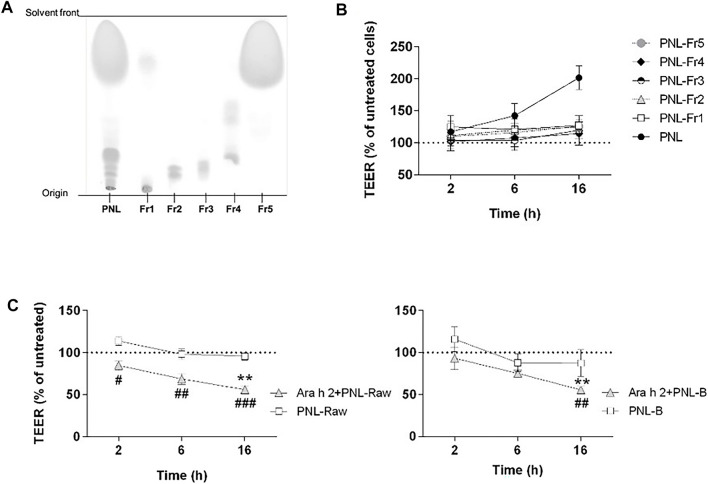
Effect of PNL fractions and PNL-preparations on the TEER of 16HBE14o- cell monolayers. **(A)** Thin-layer chromatography showing PNL and the PNL-fractions (Fr1, Fr2, Fr3, Fr4, and Fr5). **(B)** PNL and the individual PNL-fractions were applied to the apical compartments of cell monolayers and TEER was measured after 2, 6, and 16 h. Data of three experiments (mean ± SD) are presented as the percentage of the TEER value of untreated cells, indicated by a dashed line. **(C)** 16HBE14o- cell monolayers were stimulated apically with PNL isolated from raw (PNL-Raw) and boiled (PNL-B) peanuts ± Ara h 2. TEER was measured after 2, 6, and 16 h, and results are reported as percentage of TEER of untreated cells (dashed line). Data represent the mean ± SD of two experiments. *p* < 0.05, *p* < 0.01, *p* < 0.001. (#) indicates significance between Ara h 2 + PNL (Raw or B) vs. PNL (Raw or B) alone; (*) indicates significance vs. untreated control.

We next investigated whether the improvement of barrier properties upon application of PNL and GPL was the mediated by a single class of lipids within the whole extract. Hence, we fractionated the total GPL and the PNL extracts into lipid fractions corresponding to major lipid classes (Figures 5A, 6A, respectively). Each of the fractions was assayed on the cellular monolayers, and the TEER was monitored for up to 16 h (Figures 5C, 6B). None of the GPL-fractions alone showed any effect on the TEER as observed for the whole GPL extract. The same was observed for the PNL fractions PNL-Fr1-5 corresponding to the major lipid classes: none of the PNL fractions affected the permeability of the cell monolayer. As controls, we treated cells with synthetic lipids choosing oleic acid and glyceryl trioleate as representatives of free fatty acids and triacylglycerols, respectively. The synthetic lipids did not show any effect on the TEER (Supplementary Figure S4).

### 3.6 Effect of different PNL-preparations on the barrier properties of 16HBE14o-

We investigated whether peanut lipids extracted from raw (PNL-Raw) or boiled peanuts (PNL-B) affect the epithelial barrier in the same way as PNL extracted from roasted peanuts. Therefore, we treated cells apically with PNL-Raw or PNL-B at 100 μg/mL alone or in the presence of Ara h 2 (Figure 6C). Neither PNL-Raw nor PNL-B caused the changes in TEER observed with PNL. Moreover, a combination of Ara h 2 with PNL-Raw or PNL-B caused a significant drop in TEER values after 16 h (Ara h 2 + PNL-Raw or Ara h 2 + PNL-B vs. untreated *p* < 0.01).

To understand whether the differences in the effect of the various lipids preparations on epithelial cells could be explained by dissimilarities of their composition, we analyzed the three peanut oils by ^1^H-NMR spectroscopy recorded in CDCl_3_ (Supplementary Figure S3). PNL-Raw and PNL-B gave rise to identical spectra. In contrast, differences in the spectrum of PNL from roasted peanuts were found and highlighted by black arrows. One of the detectable differences was a clear broad OH peak appearing at 3.5 ppm, which corresponded to mono- or di-glyceride.

## 4 Discussion

The paradigm of food allergy pathogenesis is based on the concept that food allergen sensitization begins at the gastrointestinal mucosa after oral exposure (Burks, 2008). However, children with peanut allergy react upon their first known ingestion of peanuts, suggesting that sensitization may have occurred through a non-oral route (Bock et al., 2001). People might get exposed to peanut *via* inhalation within the household environment or at school (Brough et al., 2013; Bertelsen et al., 2014; Sheehan et al., 2017), or within the environment of an airplane (Sicherer et al., 1999). That exposure to peanuts through the skin or the airways results in sensitization has been shown in animal studies (Tordesillas et al., 2014; Wavrin et al., 2014; Dolence et al., 2018). Moreover, peanut-specific CD4^+^ T cells from peanut-allergic subjects have increased expression of the airway-homing chemokine receptor CCR4, suggesting that peanut sensitization occurred in the lungs (Blom et al., 2018). Hence, the respiratory tract is a probable site for sensitization to environmental peanuts.

Our study is the first to explore the influence of the exposure of peanut allergens and lipids on the human airway epithelium. We demonstrated for the first time that the major peanut allergens Ara h 1 and Ara h 2 impacted the barrier integrity of the 16HBE14o- bronchial epithelial cells, and they were both able to cross the epithelial barrier. In this process, the allergens maintained their antibody-binding capacities and induced the release of pro-inflammatory mediators. Additionally, we found that lipids from roasted peanuts improved the barrier function of the cell monolayers, decreased paracellular permeability, and limited the passage of peanut allergens through the epithelial layer.

We measured a decreased TEER upon application of Ara h 1 and Ara h 2 to the cellular monolayer, indicating that allergens disrupted the epithelial barrier. However, we found differences between the effects of the two allergens. Ara h 1 caused a decrease of the TEER by 40% 16 h post application and only at the highest concentration of 100 μg/mL (Figure 1). In contrast, Ara h 2 reduced the TEER by 40% just 2 h after application and the effect was maintained throughout 16 h of observation. Lower concentrations of Ara h 2 (25 and 50 μg/mL) also reduced the TEER by about 15% at the latest time point measured. To examine whether the changes seen in the TEER reflected alterations in the physical barrier integrity, we carried out a permeability assay of the monolayers using FD4, FD10, and FD70. Even though in presence of FD the allergens were still able to reduce the TEER as seen without FD, we have not measured any alteration in the concentration of FD at the basolateral side. This suggests that the drop of TEER caused by the peanut allergens (Figure 1 and Supplementary Figure S1) is not sufficient to increase paracellular permeability at any molecular weight of FD investigated (Figure 2). In contrast, we have seen that applying PNL onto the cells resulted in a decreased permeability: transfer of FD in the basolateral compartment was lower in monolayers treated with PNL + Ara h 1 and with PNL + Ara h 2 than the monolayers of untreated cells, which was in line with the increased resistance of these monolayers after 16 h. To our knowledge, the effect of PNL on the epithelium of the respiratory tract has not been studied. Our work is the first to show that PNL increase the TEER and decrease the paracellular permeability of 16HBE14o- cell monolayers.

The airways constitute a barrier to inhaled substances and the way many allergens can interact with airway epithelium has been the topic of several investigations (Lambrecht and Hammad, 2014; Van Ree et al., 2014). Certain allergens, such as Der p 1 (Wan et al., 1999), can disrupt the barrier of epithelial cells by intrinsic proteolytic activity which may facilitate allergen penetration into local tissues and enhance its interaction with the immune system; some others interact with receptors on the epithelium causing the release of Th2-associated cytokines (Hammad et al., 2009). We show that both Ara h 1 and Ara h 2 were internalized by 16HBE14o- bronchial epithelial cells and were found in the basolateral compartment, without showing colocalization with LAMP2, a marker of the late endosome/lysosome compartments (Rothen-Rutishauser et al., 2002; Schneede et al., 2011) (Figure 3A). Our data suggest a transcellular transport for Ara h 1 and Ara h 2, which was already described for both peanut allergens when exposed to intestinal epithelial cells (Price et al., 2014). About 10 ng/mL of Ara h 2 was detectable in the basolateral cell culture medium after 2 h following the apical application of 100 μg/mL of allergen (Figure 3A). A total amount of 60 ng/mL of Ara h 1 could be detected only after 16 h following treatment. The two allergens interact differently with the bronchial epithelial cells, as they also differs in size and structure: Ara h 2 is a protein of ∼17 kDa whereas Ara h 1 is a trimeric protein of about 200 kDa (Palladino and Breiteneder, 2018). However, both were detected at the basolateral side by IgE from peanut-allergic subjects, and could then potentially become available to antigen-presenting cells thus leading to allergic sensitization.

The epithelium can secrete a collection of cytokines in response to inhaled allergens that affect the immune response. Our data show that only Ara h 1 influence cytokine release. Ara h 1 increased the release of the pro-inflammatory cytokines CCL2, IL-6, and IL-8 by the bronchial epithelium (Figure 4). IL -6 was rapidly produced by the airways upon stimulation with an extract from the allergenic fungus *Aspergillus fumigatus* (Neveu et al., 2009) and together with IL-8, they were shown to attract immunocompetent cells to the barrier site following allergen exposure (Tomee et al., 1998; Röschmann et al., 2009). CCL2 is crucial for the recruitment and activation of immature monocytes, a precursors of CD11b^+^ inflammatory DCs, which can induce Th2 responses (Hammad et al., 2009; Deckers et al., 2017). The difference in behavior of Ara h 1 and Ara h 2 in regards to the release of mediators, suggest a different mechanism of interaction for each allergen with the bronchial epithelium for each allergen. However, we cannot exclude an effect of Ara h 2 at another time-points. Also, PNL do not significantly impact the production of the cytokines induced by Ara h 1. In real life, inhalation of airborne peanut particles might contain both allergens and also lipids, thus generating a different cytokine milieu compared to the one described here obtained by single allergens stimulation. How these effects translate into real life cannot be described yet using currently available experimental settings. Moreover, our study used a cell line and therefore further investigation on primary cells will be required to understand the mechanisms of allergic sensitization *via* inhalation in more detail. However, it is clear that the mechanism of sensitization is multifactorial and very complex and will need additional studies.

Besides the allergens themselves, several factors, such as lipids contained in the allergen source, contribute to the polarization of the immune system towards Th2 response and the allergic sensitization when exposed to allergenic proteins (van Wijk et al., 2005; Bublin et al., 2014; Palladino et al., 2018). To further explore the response of airway epithelial cells to peanuts, we next investigated the possible impact of PNL contained in the allergen source as a potential adjuvant in allergic sensitization. We have already shown the capacity of PNL of activating primary human keratinocytes and inducing a pro-inflammatory response (Palladino et al., 2018). PNL applied directly to the bronchial epithelial cells monolayers increased the TEER and reduced the paracellular permeability and indirectly the amount of Ara h 1 and Ara h 2 crossing the cellular monolayers. When administered with PNL, the amount of Ara h 2 transported to the basolateral side was half of the amount detected upon stimulation with Ara h 2 alone. Likewise, the concentration of Ara h 1 detected in the basolateral medium in the presence of PNL was reduced by 25%. The mechanisms by which PNL alter the transcellular transport of peanut allergens are not known and are beyond the scope of this work. Nevertheless, our data show that Ara h 1 and Ara h 2 are transported to the basolateral compartment even in the presence of PNL, however in a lower amount, which could affect the effective dose of allergen captured by DCs thus favoring the generation of a Th2 response (Constant et al., 1995).

To assess whether our findings were confined to peanuts or indicative of a more general mechanism, we sought to investigate the effect of timothy grass pollen lipids on the barrier properties of the airways epithelium. Grass pollen is one of the most common inhalant allergen sources causing IgE-mediated allergies (Bousquet et al., 2007). Lipid mediators from grass pollen can recruit and activate polymorphonuclear granulocytes (Traidl-Hoffmann et al., 2002) and eosinophils (Plötz et al., 2004). Additionally, low molecular weight components from grass pollen were shown to alter the barrier function of bronchial epithelial cells (Blume et al., 2015). In our study, we stimulated the 16HBE14o- cell monolayer with GPL and registered a strong increase of the TEER already 2 h after stimulation (Figure 5B). GPL impacted the barrier function of the 16HBE14o- bronchial epithelium in a similar way as PNL, however, their effect was greater when using equal concentrations. Ara h 2 still caused a decrease in the TEER after 2 h even in the presence of GPL.

The prevalence of peanut allergy varies between Western countries and Asia, and it has been suggested that differences in the cooking methods of peanuts are responsible for this discrepancy. In Western countries, peanuts are primarily consumed roasted, whereas in Asia peanuts are often consumed after boiling (Verhoeckx et al., 2015). It has been shown that boiling decreases the IgE binding capacity of peanut allergens whereas roasting increases peanut allergenicity and lipid-binding (Beyer et al., 2001; Mittag et al., 2004; Mondoulet et al., 2005; Nesbit et al., 2012; Maleki et al., 2014). In our experimental settings, only PNL extracted from roasted peanuts were responsible for the increased TEER of the bronchial epithelial cells. PNL-B and PNL-Raw did not elicit any response from bronchial epithelial cells and did not counteract the effect of Ara h 2 (Figure 6C). Differences in the composition of the three species were found by analysis of their ^1^H-NMR spectra (Supplementary Figure S3). While raw and boiled peanut oil have identical spectra, peanut oil from roasted peanuts presented differences in the chemical shift pattern. The ^1^H-NMR spectrum of PNL revealed the presence of mono- and di-glycerides. During the roasting process of peanuts, due to the very high temperatures a degradation of the triglyceride occurs, resulting in the formation of mono- or di-glyceride (Zhang et al., 2022). Whether these species found in the PNL from roasted peanuts, contributes to the biological effects seen in our work needs to be investigated. However, the effect of PNL and GPL on the increase of the TEER of bronchial epithelial cells was only observed when the total lipids extracts were applied to the epithelial cells (Figures 5C, 6B). Allergens and lipids are contained within the food matrix or within the pollen grain matrix: in both cases they are not delivered in a pure form, but as particles composed of various chemically different molecules. Hence, the observed effect by GPL and PNL on the barrier properties of the bronchial epithelial cells might be the result of a synergy of the various fractions that will lead to an effect only if combined. Our findings point towards a possible shared mechanism between food matrix-derived lipids and pollen grains-derived lipids in the interaction with airway epithelium and future studies will be needed to gain deeper insight into this topic.

Our work contributes to providing data about the effect of peanut allergens and lipids on the respiratory epithelium, which might represent a sensitizing route to peanuts. We demonstrated for the first time that the clinically relevant peanut allergens Ara h 1 and Ara h 2 cross the bronchial epithelial barrier in an immunologically active form and induce the production of pro-inflammatory cytokines by the bronchial epithelium. We also showed that PNL improve the barrier function of the bronchial epithelium and decrease the barrier permeability, thus modifying the amount of allergen reaching the basolateral side. Furthermore, we have also described a similar effect for grass pollen grains-derived lipids on the barrier properties of the bronchial epithelial cells, thus suggesting a potential shared mechanism between food matrix-derived lipids and pollen grains-derived lipids in the interaction with the airway epithelial cells.

## Data Availability

The original contributions presented in the study are included in the article/Supplementary Material, further inquiries can be directed to the corresponding author.

## Supplementary material

SUPPLEMENTARY FIGURE S1 TEER measurement in the presence of dextran. 16HBE14o- cell monolayers were treated apically with FITC-dextran (FD) of different sizes (4, 10, or 70 kDa) in the presence of allergens and/or PNL. H_2_O_2_ and EGTA were used as positive controls. TEER was monitored at 2, 6, and 16 h following treatment. The TEER of unstimulated cells treated with each FD (control) was used as a baseline and is showed by a dashed line. Data shown are the mean ± SD of three independent experiments.

SUPPLEMENTARY FIGURE S2 Paracellular permeability assay. Transport of FD4, FD10, or FD70 through the cell monolayer, in presence of EGTA and H_2_O_2_. Data (mean ± SD of three experiments) show levels of FD after 2, 6, and 16 h of apical exposure.

SUPPLEMENTARY FIGURE S3 Cytokines release upon stimulation with Ara h 2 with and without PNL. Cell monolayers were treated apically with Ara h 2 ± PNL, and the concentrations of CCL2, IL-6, and IL-8 released into the basolateral medium were measured after 16 h.

SUPPLEMENTARY FIGURE S4 Synthetic lipids do not affect TEER measurements. Glyceryl trioleate and oleic acid were added apically to 16HBE14o- cells and the TEER was measured after 2, 6, and 16 h of treatment. Results are expressed as the percentage of the TEER measured in untreated cells (dashed line). Data represent the mean ± SD of two experiments.

SUPPLEMENTARY FIGURE S5
^1^H-NMR spectra of roasted (above), raw (middle), and boiled (below) peanut lipids. Black arrows highlight major differences in the spectra.
