# Supplementary figures and images for: Peanut lipids influence the response of bronchial epithelial cells to the peanut allergens Ara h 1 and Ara h 2 by decreasing barrier permeability
Source: Front Mol Biosci. 2023 Feb 8;10:1126008. doi: 10.3389/fmolb.2023.1126008 (PMC9945344; doi:10.3389/fmolb.2023.1126008)

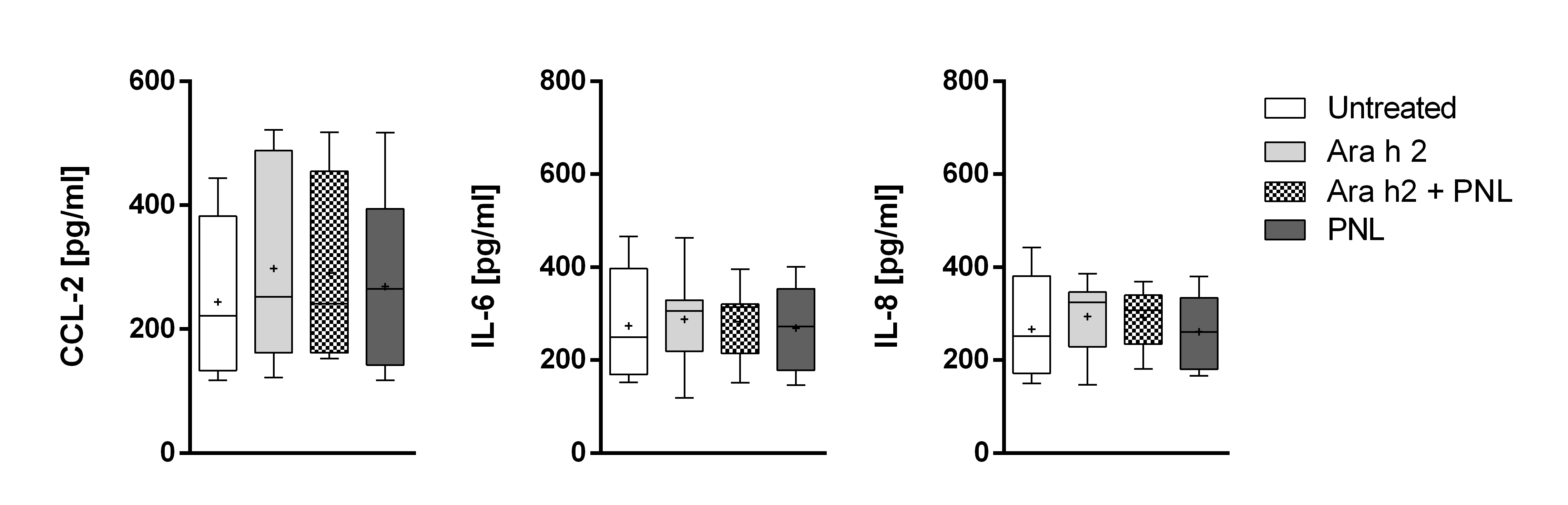

Supplement: Supplementary file 1 [file Image3.JPEG]

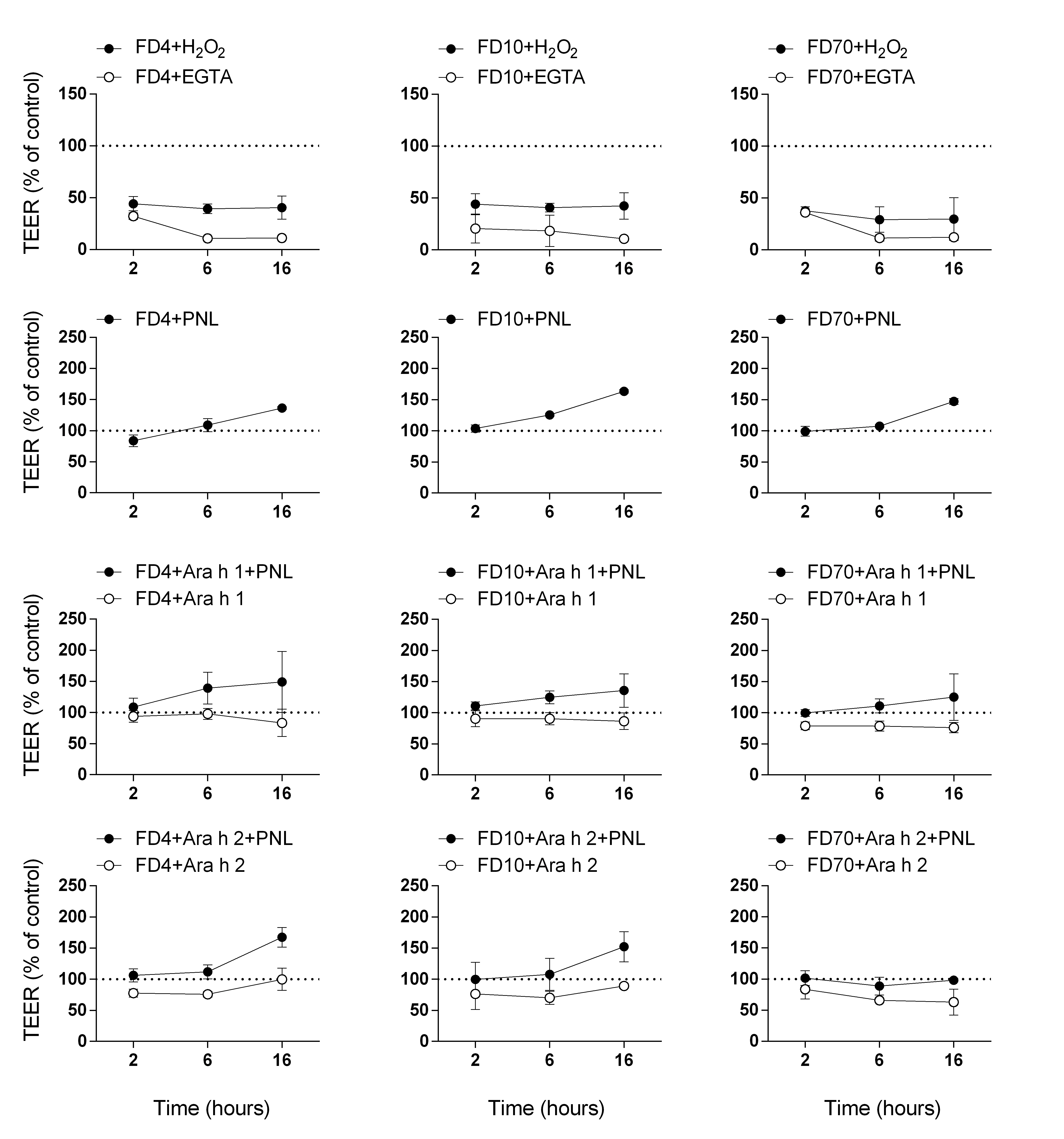

Supplement: Supplementary file 2 [file Image1.JPEG]

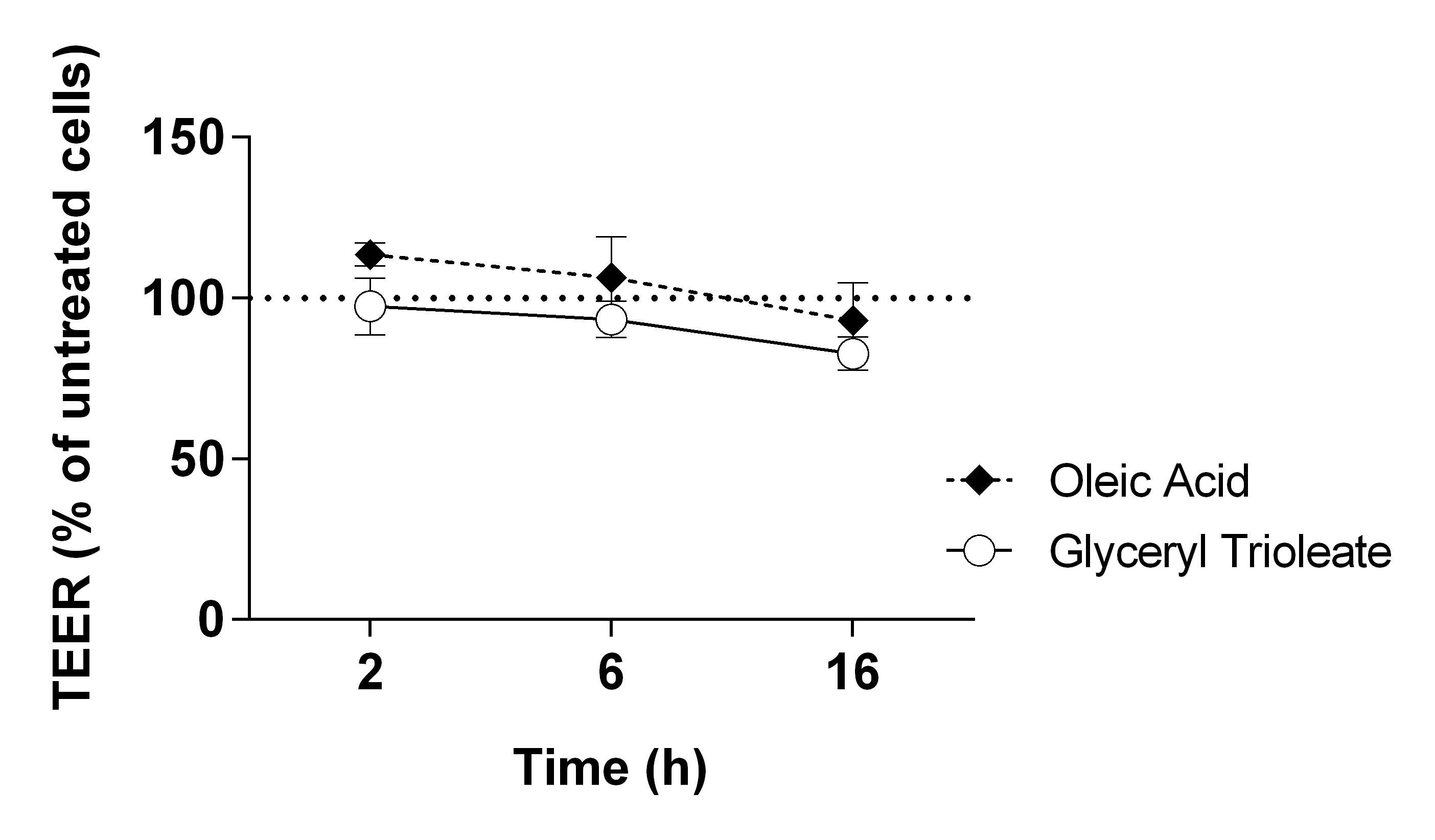

Supplement: Supplementary file 3 [file Image4.JPEG]

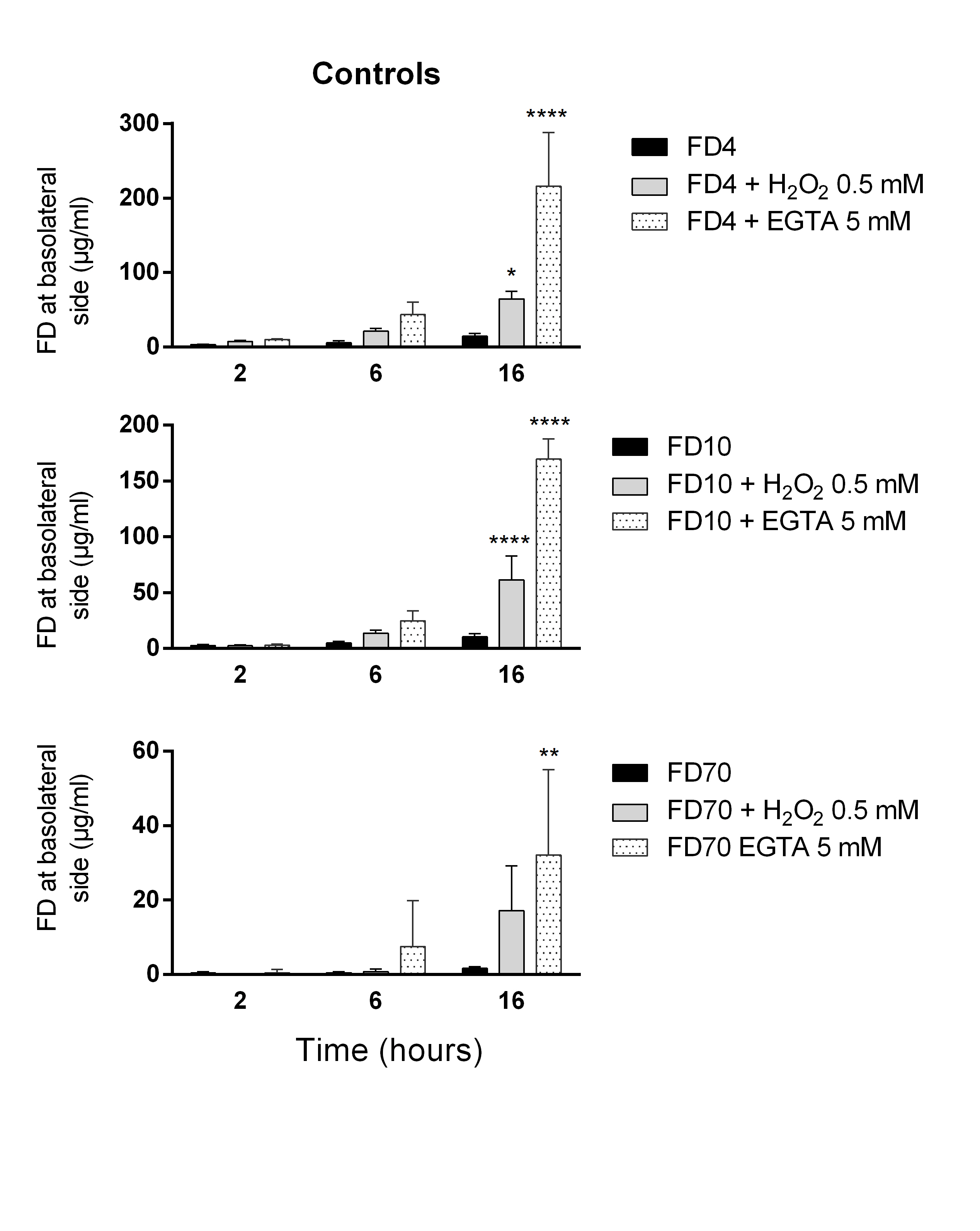

Supplement: Supplementary file 4 [file Image2.JPEG]

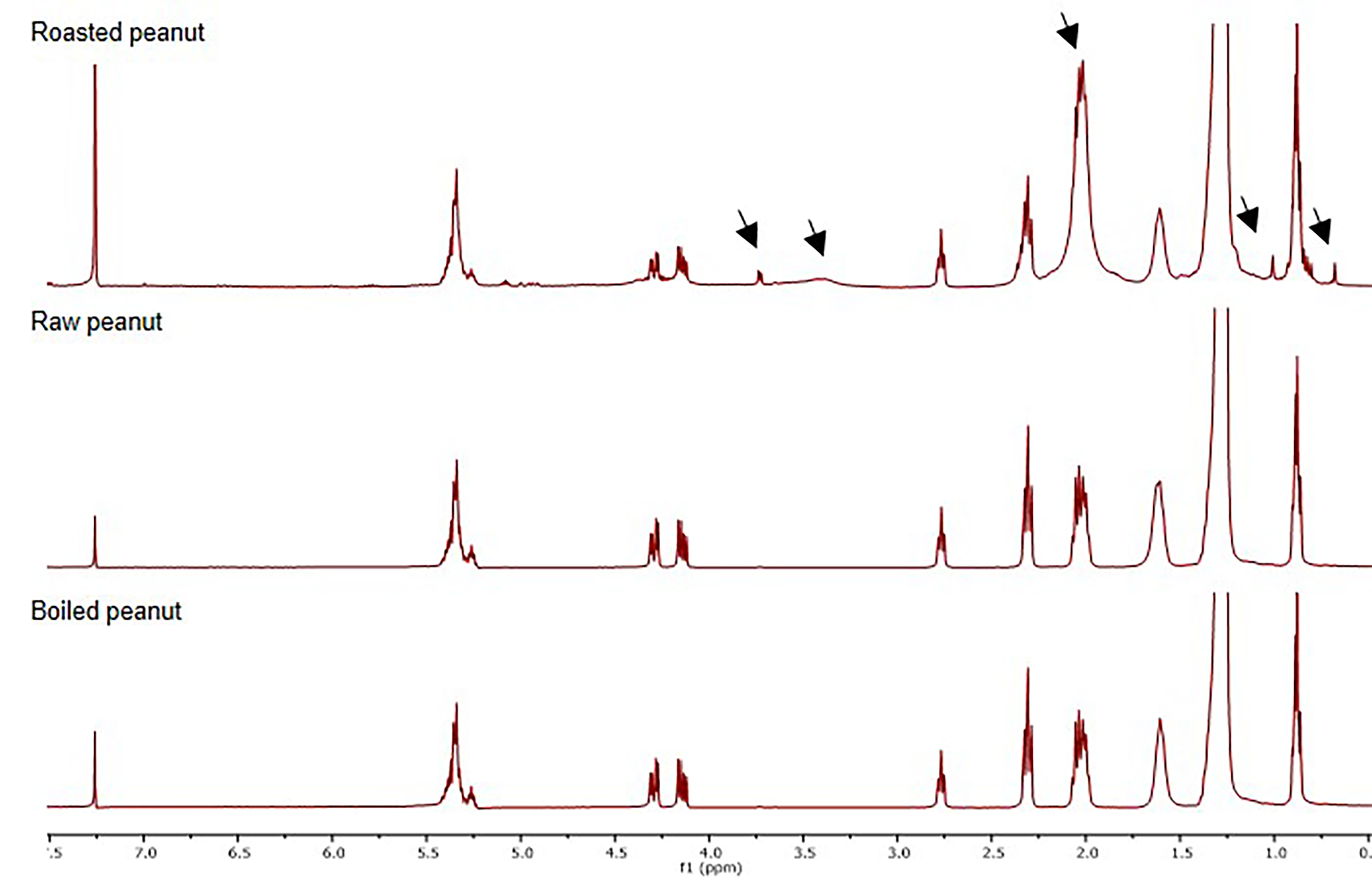

Supplement: Supplementary file 5 [file Image5.JPEG]
